# Aqueous humor monocyte chemoattractant protein-1 predicted long-term visual outcome of proliferative diabetic retinopathy undergone intravitreal injection of bevacizumab and vitrectomy

**DOI:** 10.1371/journal.pone.0248235

**Published:** 2021-03-05

**Authors:** Jianqin Lei, Guolong Ding, Anming Xie, Yaguang Hu, Ning Gao, Xiaojuan Fan

**Affiliations:** 1 Department of Ophthalmology, 1^st^ affiliated hospital of Xi’an Jiaotong University, Xi’an, Shaanxi, China; 2 Department of Ophthalmology, Xi’an No. 1 hospital, Xi’an, China; Boston University School of Medicine, UNITED STATES

## Abstract

**Purpose:**

We aim to investigate the risk factors associated with the prognosis of proliferative diabetic retinopathy (PDR) after a sequential treatment of intravitreal injection of bevacizumab (IVB) and pars plana vitrectomy (PPV).

**Methods:**

In this cohort study, 63 eyes from 55 patients (21 females) diagnosed with PDR, who needed PPV for non-clearing vitreous hemorrhage or fibrovascular membrane proliferation were enrolled. All the eyes underwent IVB followed by PPV. Anterior chamber tap was performed at the beginning of both procedures to evaluate the concentration of vascular endothelial growth factor (VEGF), interleukin (IL)-6, IL-8, and monocyte chemoattractant protein (MCP)-1.

**Results:**

Forty-seven patients (54 eyes) were followed over six months, averaging 12±5 (6–19) months. The concentration of VEGF significantly decreased after IVB (P<0.001), while other cytokines did not change significantly. The aqueous humor level of IL-8 after IVB (R = 0.378, P = 0.033), MCP-1 before (R = 0.368, P = 0.021) and after (R = 0.368, P = 0.038) IVB, and combined phacoemulsification (R = 0.293, P = 0.032) was correlated with the logMAR visual acuity at the last follow-up. Multivariate analysis showed that MCP-1 was the predictor for a worse visual outcome (B = 0.108, 95% CI 0.013–0.202; P = 0.027).

**Conclusions:**

MCP-1 was a predictor for the unfavorable visual outcome of PDR after IVB pretreatment and PPV.

## Introduction

Diabetic retinopathy is the leading cause of irreversible blindness in the working population worldwide. Pars plana vitrectomy (PPV) remains a useful cost-utility strategy for the treatment of proliferative diabetic retinopathy (PDR) [1]. However, visual prognosis is often compromised after PPV due to the high prevalence of postsurgical complications. The overall rate of postsurgical complications was reported to be as high as 30% in a recent clinical trial with pretreatment of bevacizumab [2]. The mechanism of PDR progression has not been fully understood, yet, abnormal angiogenesis and inflammation might be the essential pathogenesis.

The progression of PDR was reported to be from 25% to 32% after PPV and was associated with a high intravitreal concentration of vascular endothelial growth factor (VEGF) [3,4]. Recurrent vitreous hemorrhage (RVH) is one of the most common postsurgical complications for PDR, with a reported incidence of up to 75% [5]. Also, RVH has been demonstrated to be correlated with the increased intraocular concentration of VEGF [6].

Apart from VEGF, certain inflammatory cytokines including interleukin (IL)-6, IL-8, and monocyte chemoattractant protein (MCP)-1 were also found to have a possible connection with PDR progression after PPV [4,7,8]. However, those studies were based on PPV alone, without the pretreatment of anti-VEGFs. In the recent decade, the application of anti-VEGFs before PPV for PDR has been proved to facilitate the surgery and associated with less early vitreous hemorrhage [9]. And there are clues that intravitreal injection of anti-VEGFs can change not only the intraocular level of VEGF but also certain inflammatory cytokines [10,11]. It is not fully understood whether anti-VEGFs could change the influence of VEGF or other inflammatory cytokines on the prognosis of PDR.

In the present study, we aim to investigate the possible factors that could be associated with an unfavorable visual outcome or RVH after PPV and pretreatment of bevacizumab for PDR.

## Materials and methods

### Data collection

In this clinical cohort study, all the participants were recruited from one center (Ophthalmology department of Xi’an Jiaotong University) from March, 2014 to March, 2015, and the following data collection was terminated by December, 2015. The inclusion criteria are 1) Patients were diagnosed of PDR in at least one eye. 2) Patients were suggested by their attending doctors to undergo intravitreal injection of bevacizumab (IVB) and PPV due to unclearing vitreous hemorrhage and/or vision threatening fibrovascular membrane. For each patient, the age, gender, type of diabetes, duration of visual impairment, and the history of previous surgery or scattered photocoagulation was registered at the enrollment. Routine ophthalmic examinations, color fundus photographs, and serum levels of glycosylated hemoglobin (HbAc1) and creatinine were obtained within a week before surgeries. The enrolled participants were asked to follow up at 1 week, 1 month and then every 3 months after surgery with a minimum follow-up requirement of 6 months. Routine ophthalmic examinations, including best corrected visual acuity (BCVA), intraocular pressure, slit lamp biomicroscopy, and mydriatic stereoscopic fundus examination were required at each visit, however, optical coherence tomography angiography (OCT) was not a mandate. The participants were excluded if vision threatening retinopathies other than diabetic retinopathy were confirmed (for example, macular hole, pathological myopia, and choroidal neovascularization). The study was approved by the Institutional Review Board of the 1^st^ affiliated hospital of Xi’an Jiaotong University and conducted in accordance with the ethical standards stated in the Declaration of Helsinki. Written informed consent was obtained from all individuals before study participation.

### Surgical procedures

All patients had received intravitreal injection of 1.25mg/0.05ml bevacizumab 3 to 7 days before a standard 23-gauge pars plana vitrectomy (PPV). If there’s cataract that interfered with posterior segment visualization, phacoemulsification and intraocular lens implantation was combined with the PPV. The contact lens was used for viewing during the surgeries. Electric diathermy was used where necessary and pan-retinal photocoagulation was completed during the surgery if possible. Whether a silicone oil tamponade was performed was up to the surgeon’s decision. All PPV surgeries were performed by two doctors (J.L. & A.X.). During the follow-up, complementary PRP was suggested if necessary. Repeated intravitreal injection of bevacizumab was also suggested if center involved macular edema was evidenced. And these decisions were also made by either of the two doctors.

### Sample collection

Aqueous humor sample of at least 50μl was obtained at the start of the intravitreal injection and the PPV, respectively. The Samples were instilled into capped tubes and stored at -80°C until analyzed. The concentration of IL-6, IL-8, MCP-1, and VEGF in the aqueous humor was measured using cytometric beads array in the central laboratory of our hospital.

### Outcome measurements and statistics

BCVA was obtained using the Snellen Chart and was transformed into LogMAR visual acuity. The logMAR visual acuity at the last follow-up and whether there’s post-surgical recurrent vitreous hemorrhage (RVH) was used as two outcome measurements. RVH was defined as postsurgical vitreous haze from red blood cells that prevented viewing the fundus. Small amount of red blood cells that appeared immediately after surgery and were cleaned around 1 week was not considered as RVH. Paired sample T-test was used to compare the level of cytokines in the aqueous humor before and after IVB, and so was the comparison between the BCVA before the operation and at the last follow-up. An independent sample T-test was used to compare the level of aqueous humor cytokines between the combined cataract surgery group and PPV alone group. Aqueous humor cytokine levels and other clinical factors, including age, gender, level of glycosylated hemoglobin, increased serum creatinine, duration of visual impairment, previous PRP, previous pseudo-phakic eye, presence of tractional retinal detachment (TRD), the combination of phacoemulsification and intraocular lens implantation, and combination of silicone oil tamponade were evaluated regarding the association with the outcome measurements of the last follow-up using Pearson or Spearman’s rho correlations. In the multivariate analysis, the aqueous humor levels of cytokines after IVB were excluded from the candidate risk factors. A linear regression model was used for evaluating the predictors of final visual outcome and a binary logistic regression model was applied to analyze the predictors of post-surgical RVH. The missing data was not included in the analysis. The software SPSS (version 19.0. IBM corporation) was used for all the statistics. The sample size was estimated using software PASS (version 11.0.7. NCSS corporation)

## Results

### Descriptions of the sample

Fifty-five patients (21 female), 63 eyes were enrolled in the study. Averaged age was 54±12 (29–79) years. Among them, 3 patients (3 eyes) were diagnosed with type 1 diabetes and the rest were all type 2. Although all the patients were asked to visit back according to a timetable, it turned out that the majority of them didn’t follow it. The patients tended not to come back regularly until visual loss. Thus, only the outcome measurements of the last follow-up that was no less than six months were obtained. By December, 2015, 47 patients (54 eyes) were followed over six months. The period of follow-up was 12±5 (6–19) months. Four (4 eyes) patients lost contact and 4 patients (5 eyes) died before an ophthalmic examination could be achieved no less than 6 months. Pre-operatively, the average level of HbAc1 was 7.5±1.8 (4.5–13.6) % (N = 50) and 47.6% (30/63) had abnormally increased creatinine level (>97μmol/L). Thirty-seven out of 63 eyes (58.7%) had previous scattered photocoagulations. The proportion of pseudo-phakic eyes was 12.7% (8/63). Among the 55 phakic eyes, 14 (25%) had combined phacoemulsification and IOL implantation during the PPV. Tractional retinal detachment was diagnosed in 36.5% (23/63) of the eyes before or during the surgery. Fourteen out of 63 eyes (22.2%) had combined silicone oil tamponade.

### Cytokine levels in aqueous humor

**Fig 1:**
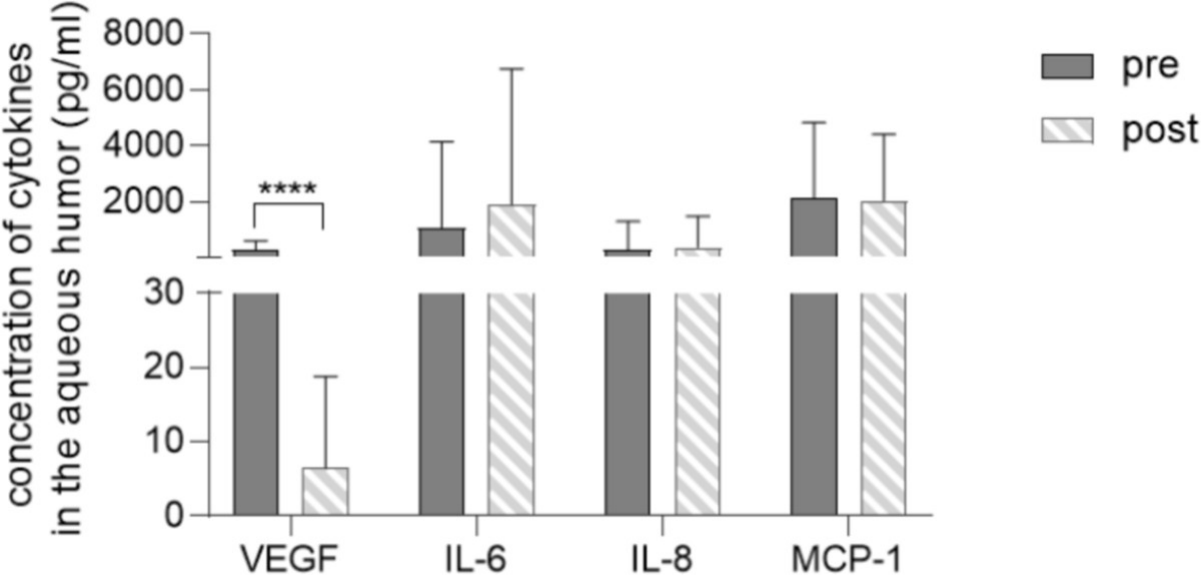
Level of cytokine pairs in the aqueous humor before and after intravitreal injection of bevacizumab. (****: P<0.0001).

We had successfully acquired aqueous humor samples from 46 eyes before IVB and from 37 eyes at the beginning of the PPV. The average concentration of VEGF, IL-6, IL-8 and MCP-1 was 250.7±299.4 (3.4–1673.0), 2993.2±11609.8 (3.98–74057.7), 258.7±848.3 (2.24–5737.5) and 2135.1±2434.2 (295.2–14806.4) pg/ml before IVB, and 7.6±15.1 (0.0–70.8), 2025.5±4867.2 (7.1–22453.9), 365.1±1022.8 (14.4–6282.7) and 2136.6.1±2277.1 (401.4–12477.3) pg/ml before vitrectomy. The aqueous humor sample was acquired before both IVB and PPV in only 32 eyes. The concentration of VEGF was significantly decreased after IVB (P<0.001), while other cytokines did not change significantly (Fig 1). Independent T-test showed the levels of IL-6, IL-8, and MCP-1 were higher in the combined cataract surgery group than that in the PPV alone group, however, the differences were not statistically significant.

### Visual outcome

The average logMAR BCVA before surgery and at the last follow-up were 1.70±0.49 and 0.93±0.71, respectively and the difference is statistically significant (P<0.001). Among the 54 followed eyes, 22 (40.7%) eyes could achieve a final visual acuity of no less than 20/60. The correlation between the level of aqueous humor cytokines and the final visual outcome was shown in Table **1**. The univariate analysis revealed that the aqueous humor level of IL-8 after IVB (R = 0.378, P = 0.033), MCP-1 before IVB (R = 0.368, P = 0.021) and MCP-1 after IVB (R = 0.368, P = 0.038) were positively correlated with the logMAR BCVA at the last follow-up. The correlation between the clinical factors and the final visual outcome was displayed in Table **2**. Combined phacoemulsification was positively correlated with the logMAR BCVA at the last follow-up (R = 0.293, P = 0.032). Postsurgical recurrent vitreous hemorrhage was also associated with worse visual outcomes (R = 0.468, P<0.001). The rest factors had no significant correlation. The multivariate analysis (stepwise) showed that only MCP-1 was the predictor for the worse visual prognosis (B = 0.108, 95% CI 0.013–0.202; P = 0.027). If the aqueous humor level of MCP-1 increased by 1 ng/ml, the final visual outcome would be worse by 0.1 logMAR visual acuity.

**Table 1:** The correlation between the level of aqueous humor cytokines and the outcome measurements.

Aqueous humor level of cytokines	logMAR BCVA at the last follow-up		Post-surgical recurrent vitreous hemorrhage	
	R value	P value	R value	P value
VEGF before IVB	0.038	0.816	0.070	0.671
VEGF before PPV	0.076	0.679	0.161	0.380
IL-6 before IVB	-0.068	0.681	0.027	0.870
IL-6 before PPV	0.146	0.425	0.151	0.408
IL-8 before IVB	0.302	0.062	-0.062	0.707
IL-8 before PPV	0.378	0.033	0.282	0.117
MCP-1 before IVB	0.368	0.021	-0.043	0.794
MCP-1 before PPV	0.368	0.038	0.172	0.347

BCVA- best corrected visual acuity.

IL- interleukin.

IVB- intravitreal injection of bevacizumab.

MCP- monocyte chemoattractant protein.

PPV- pars plana vitrectomy.

VEGF- vascular endothelial growth factor.

**Table 2:** The correlation between the clinical factors and the outcome measurements.

Clinical factors	logMAR BCVA at the last follow-up		Post-surgical recurrent vitreous hemorrhage	
	R value	P value	R value	P value
Age (years)	0.060	0.665	-0.049	0.725
gender	-0.075	0.592	-0.106	0.441
Type of diabetes	0.065	0.640	-0.067	0.627
Duration of visual impairment (months)	-0.024	0.863	0.135	0.326
HbA1c (%)	-0.009	0.955	0.130	0.399
logMAR BCVA before surgery	0.224	0.104	-0.060	0.662
Creatinine >97μmol/L	0.085	0.540	-0.040	0.771
pseudo-phakic eyes	-0.192	0.165	-0.202	0.140
combined phacoemulsification	0.293	0.032	-0.066	0.633
previous scattered photocoagulation	-0.141	0.310	0.053	0.702
Tractional retinal detachment	0.250	0.068	0.382	0.004
Combined silicone oil tamponade	0.209	0.129	0.254	0.061
Post-surgical recurrent vitreous hemorrhage	0.468	<0.001		

BCVA- best corrected visual acuity.

### Post-surgical vitreous hemorrhage

Twelve out of 55 eyes (1 case lost follow-up after recurrent vitreous hemorrhage) (22%) had recurrent vitreous hemorrhage. Among the 12 eyes, RVH occurred within 1 week after surgery in 8 eyes, at month 2 in 1 eye, at month 3 in two eyes and at month 6 in 1 eye. The correlation between aqueous humor cytokines and postsurgical RVH was shown in Table 1. None was found to have a significant correlation. In Table 2, the correlation between clinical factors and postsurgical RVH was listed. And TRD was found to correlate with RVH (R = 0.382, P = 0.004).

### Other adverse events

Three eyes developed neovascular glaucoma and 1 eye had retinal detachment after PPV. And all the 4 eyes had RVH. Four patients died before an ophthalmic examination could be achieved no less than six months post-operatively. Among them, 1 (two eyes enrolled) died of multiple organ failure, 1 died of stroke, and 2 died of renal failure, at week 2, month 2, month 12 and month 13, respectively. Another two patients died (one from pulmonary fibrosis at month 7 and the other from heart attack at month 18) after they had a final ophthalmic examination over six months, so these two were not excluded from the study.

## Discussion

Previously, several studies have investigated the relationship between intraocular cytokines and the prognosis of PDR after PPV. Funatsu et al. [3,4] initially suggested that the vitreous level of VEGF was a predictor for PDR progression after vitrectomy and the vitreous level of IL-6 was higher in the progression group. Later, Wakabayashi et al. [6] also reported that increased intraocular VEGF was associated with early vitreous hemorrhage and neovascular glaucoma after vitrectomy. Besides, Suzuki et al. [12] demonstrated that intravitreal VEGF level was related to the progression of PDR after PPV in a larger cohort. Apart from VEGF, some inflammatory cytokines might also play an important role. Petrovic et al. [13] proposed that the vitreous level of IL-8 predicted poor visual outcomes. Recently, Yoshida et al. [8] suggested that the intraocular level of MCP-1, IL-6, and IL-8 might be the cause of fibrous proliferation and reoperation for PDR. Different from the above studies, the patients in our cohort all underwent a pretreatment of anti-VEGFs before PPV, and it turned out that the intraocular level of VEGF, whether prior or post anti-VEGF treatment, had no significant correlation with the visual outcome or postsurgical vitreous hemorrhage. Thus, we suppose that the prognosis of PDR after PPV was no longer influenced by the intraocular VEGF level with the combined use of anti-VEGFs. On the other hand, the aqueous humor level of MCP-1 and post-IVB level of IL-8 was negatively correlated with visual outcomes. The levels of these two cytokines were not inhibited by the anti-VEGFs, neither was their influence on the visual prognosis. In some studies, the short-term aqueous level of IL-8 even increased after intravitreal injection of bevacizumab [11,14].

IL-8 is one of the major mediators of the inflammatory response. It is secreted by several cell types and functions as a chemoattractant and as a potent angiogenic factor. Recently, Wu et al. [15] reported that IL-8 demonstrated a strong correlation in vitreous and aqueous of patients with PDR and recent anti-VEGF injection did not significantly affect IL-8. In the univariate analysis of our study, aqueous IL-8 was correlated with a worse visual prognosis probably because of its role in fibrous proliferation.

In particular, our present study first revealed that MCP-1 was a predictor for the visual prognosis of PDR patients after IVB and PPV. MCP-1 is a member of the CC chemokine family that stimulates the recruitment and activation of monocytes and macrophages which could lead to higher microvascular permeability or ischemia from vessel occlusions [16]. Many previous studies have demonstrated the close relationship between MCP-1 and the severity of diabetic retinopathy [17–19]. Yoshida et al. [7] had reported the association between MCP-1 and diabetic macular edema after PPV. There’s also evidence that the MCP-1 concentration was markedly elevated after PPV, implying an association between the prolonged inflammation after vitrectomy and complications, especially TRD [20]. We suppose that the inflammatory reaction induced by high MCP-1 might aggravate macular edema or progression of PDR, which could compromise postsurgical visual function. Some pilot studies have investigated in the effect of intravitreal triamcinolone [21] or dexamethasone implant [22,23] on the intraocular inflammatory cytokines and both drugs could significantly decrease the level of MCP-1 in the aqueous humor. A further clinical study is warranted to evaluate the effect of combined intraocular steroids during PPV on the prognosis of PDR.

Interestingly, we also found that combined phacoemulsification during PPV was associated with worse visual outcomes. We suppose that the combined surgery might well enhance the intraocular inflammatory cytokines, which could lead to a less optimal visual outcome. However, the intraocular inflammatory cytokines could be higher in PDR eyes with more severe cataract, thus, the probability of existed macular damage could be higher. Although the levels of inflammatory cytokines in the combined cataract surgery group were a bit higher, the differences were not statistically significant in this study. Previously, a single-center unrandomized clinical study had compared the combined surgery and a two-step surgical procedure for PDR. It turned out that sequential surgery could be advantageous to BCVA outcomes by minimizing postoperative VH, which is significantly more frequent after combined surgery [24].

In the present study, postsurgical VH was no longer associated with high levels of intraocular VEGF with preoperative bevacizumab intravitreal injection. Rather, RVH was associated with tractional retinal detachment. In such a condition, preretinal fibrovascular proliferation is usually more severe, which could lead to recurrence of hemorrhage after surgery. We believe that proper intraocular diathermy would be more essential in the prevention of RVH after PPV in PDR eyes.

Our study has several limitations which should be considered in assessing the significance of this study. Although this cohort included 63 eyes, around 14% lost follow-up over six months. Besides, aqueous humor acquisition was not achieved in all the included eyes, which led to smaller sample size. Another limitation is that the period of follow-up was not consistent and a time-to-event analysis was not used due to the limited sample size. However, since the event such as vision or RVH did not seem to be correlated with the duration after surgery, specifying the endpoint as over six months was fairly acceptable. Besides, unfortunately, we did not achieve OCT images consistently at the last follow-up, otherwise, the influence of cytokines on the retinal structure would have been assessed.

Despite those limitations, this study still showed some clues of the influence of certain inflammatory cytokines on the prognosis of PDR. In summary, our study first revealed that aqueous humor MCP-1 was a predictor for the worse visual outcome of PDR after vitrectomy, while the level of aqueous humor VEGF was associated with neither the postsurgical visual outcome nor the RVH after a sequential procedure of anti-VEGF intravitreal injection and PPV. In addition, combined phacoemulsification plus IOL implantation with PPV was probably associated with a less optimal visual outcome.

## Supporting information

S1 Checklist STROBE statement—checklist of items that should be included in reports of observational studies. (DOC)

S1 Raw data (XLSX)

## References

[1] Lin J, Chang JS, Yannuzzi NA, Smiddy WE. Cost Evaluation of Early Vitrectomy versus Panretinal Photocoagulation and Intravitreal Ranibizumab for Proliferative Diabetic Retinopathy. Ophthalmology. 2018;125: 1393–1400. 10.1016/j.ophtha.2018.02.038 29606379 PMC6941652

[2] Castillo J, Aleman I, Rush SW, Rush RB. Preoperative Bevacizumab Administration in Proliferative Diabetic Retinopathy Patients Undergoing Vitrectomy: A Randomized and Controlled Trial Comparing Interval Variation. Am J Ophthalmol. 2017;183: 1–10. 10.1016/j.ajo.2017.08.013 28860046

[3] Funatsu H, Yamashita H, Noma H, Mimura T, Sakata K, Hori S. Risk evaluation of outcome of vitreous surgery for proliferative diabetic retinopathy based on vitreous level of vascular endothelial growth factor and angiotensin II. Br J Ophthalmol. 2004;88: 1064–8. 10.1136/bjo.2003.032656 15258026 PMC1772284

[4] Funatsu H, Yamashita H, Mimura T, Noma H, Nakamura S, Hori S. Risk evaluation of outcome of vitreous surgery based on vitreous levels of cytokines. Eye (Lond). 2007;21: 377–82. 10.1038/sj.eye.6702213 16410812

[5] Oyakawa RT, Schachat AP, Michels RG, Rice TA. Complications of Vitreous Surgery for Diabetic Retinopathy: I. Intraoperative Complications. Ophthalmology. 1983;90: 517–521. 10.1016/s0161-6420(83)34526-7 6877782

[6] Wakabayashi Y, Usui Y, Okunuki Y, Ueda S, Kimura K, Muramatsu D, et al. Intraocular VEGF level as a risk factor for postoperative complications after vitrectomy for proliferative diabetic retinopathy. Invest Ophthalmol Vis Sci. 2012;53: 6403–10. 10.1167/iovs.12-10367 22899753

[7] Yoshida S, Kubo Y, Kobayashi Y, Zhou Y, Nakama T, Yamaguchi M, et al. Increased vitreous concentrations of MCP-1 and IL-6 after vitrectomy in patients with proliferative diabetic retinopathy: possible association with postoperative macular oedema. Br J Ophthalmol. 2015;99: 960–6. 10.1136/bjophthalmol-2014-306366 25631486

[8] Yoshida S, Kobayashi Y, Nakao S, Sassa Y, Hisatomi T, Ikeda Y, et al. Differential association of elevated inflammatory cytokines with postoperative fibrous proliferation and neovascularization after unsuccessful vitrectomy in eyes with proliferative diabetic retinopathy. Clin Ophthalmol. 2017;11: 1697–1705. 10.2147/OPTH.S141821 29033535 PMC5614779

[9] Zhao X, Xia S, Chen Y. Antivascular endothelial growth factor agents pretreatment before vitrectomy for complicated proliferative diabetic retinopathy: a meta-analysis of randomised controlled trials. Br J Ophthalmol. 2018;102: 1077–1085. 10.1136/bjophthalmol-2017-311344 29246890 PMC6059039

[10] Suzuki Y, Suzuki K, Yokoi Y, Miyagawa Y, Metoki T, Nakazawa M. Effects of intravitreal injection of bevacizumab on inflammatory cytokines in the vitreous with proliferative diabetic retinopathy. Retina. 2014;34: 165–71. 10.1097/IAE.0b013e3182979df6 23851630

[11] Forooghian F, Kertes PJ, Eng KT, Agrón E, Chew EY. Alterations in the intraocular cytokine milieu after intravitreal bevacizumab. Invest Ophthalmol Vis Sci. 2010;51: 2388–92. 10.1167/iovs.09-4065 20007836 PMC2868488

[12] Suzuki Y, Suzuki K, Kudo T, Metoki T, Nakazawa M. Level of Vascular Endothelial Growth Factor in the Vitreous Fluid of Proliferative Diabetic Retinopathy Patients and Prognosis after Vitrectomy. Ophthalmologica. 2016;236: 133–138. 10.1159/000449261 27794575

[13] Petrovič MG, Korošec P, Košnik M, Hawlina M. Association of preoperative vitreous IL-8 and VEGF levels with visual acuity after vitrectomy in proliferative diabetic retinopathy. Acta Ophthalmol. 2010;88: e311–6. 10.1111/j.1755-3768.2010.02030.x 21073666

[14] Jeon S, Lee WK. Intravitreal bevacizumab increases intraocular interleukin-6 levels at 1 day after injection in patients with proliferative diabetic retinopathy. Cytokine. 2012;60: 535–9. 10.1016/j.cyto.2012.07.005 22846147

[15] Wu F, Phone A, Lamy R, Ma D, Laotaweerungsawat S, Chen Y, et al. Correlation of aqueous, vitreous, and plasma cytokine levels in patients with proliferative diabetic retinopathy. Investig Ophthalmol Vis Sci. 2020;61: 26. 10.1167/iovs.61.2.26 32084272 PMC7326572

[16] Taghavi Y, Hassanshahi G, Kounis NG, Koniari I, Khorramdelazad H. Monocyte chemoattractant protein-1 (MCP-1/CCL2) in diabetic retinopathy: latest evidence and clinical considerations. J Cell Commun Signal. 2019; 13: 451–462. 10.1007/s12079-018-00500-8 30607767 PMC6946768

[17] Hernández C, Segura RM, Fonollosa A, Carrasco E, Francisco G, Simó R. Interleukin-8, monocyte chemoattractant protein-1 and IL-10 in the vitreous fluid of patients with proliferative diabetic retinopathy. Diabet Med. 2005;22: 719–22. 10.1111/j.1464-5491.2005.01538.x 15910622

[18] Zhou J, Wang S, Xia X. Role of intravitreal inflammatory cytokines and angiogenic factors in proliferative diabetic retinopathy. Curr Eye Res. 2012;37: 416–20. 10.3109/02713683.2012.661114 22409294

[19] Reddy S, Amutha A, Rajalakshmi R, Bhaskaran R, Monickaraj F, Rangasamy S, et al. Association of increased levels of MCP-1 and cathepsin-D in young onset type 2 diabetes patients (T2DM-Y) with severity of diabetic retinopathy. J Diabetes Complications. 2017;31: 804–809. 10.1016/j.jdiacomp.2017.02.017 28336215

[20] Sassa Y, Yoshida S, Ishikawa K, Asato R, Ishibashi T, Kono T. The kinetics of VEGF and MCP-1 in the second vitrectomy cases with proliferative diabetic retinopathy. Eye (Lond). 2016;30: 746–53. 10.1038/eye.2016.20 26915745 PMC4869133

[21] Sohn HJ, Han DH, Kim IT, Oh IK, Kim KH, Lee DY, et al. Changes in aqueous concentrations of various cytokines after intravitreal triamcinolone versus bevacizumab for diabetic macular edema. Am J Ophthalmol. 2011;152: 686–94. 10.1016/j.ajo.2011.03.033 21782151

[22] Podkowinski D, Orlowski-Wimmer E, Zlabinger G, Pollreisz A, Mursch-Edlmayr A-S, Mariacher S, et al. Aqueous humour cytokine changes during a loading phase of intravitreal ranibizumab or dexamethasone implant in diabetic macular oedema. Acta Ophthalmol. 2020; 98: e407–e415. 10.1111/aos.14297 31736269 PMC7318694

[23] Figueras-Roca M, Sala-Puigdollers A, Zarranz-Ventura J, Alba-Linero C, Alforja S, Esquinas C, et al. Anatomic response to intravitreal dexamethasone implant and baseline aqueous humor cytokine levels in diabetic macular edema. Investig Ophthalmol Vis Sci. 2019;60: 1336–1343. 10.1167/iovs.18-26215 30933261

[24] Rivas-Aguiño P, García-Amaris RA, Berrocal MH, Sánchez JG, Rivas A, Arévalo JF. [Pars plana vitrectomy, phacoemulsification and intraocular lens implantation for the management of cataract and proliferative diabetic retinopathy: comparison of a combined versus two-step surgical approach]. Arch Soc Esp Oftalmol. 2009;84: 31–8. 10.4321/s0365-66912009000100005 19173136

